# TNF-α inhibitors for type 1 diabetes: exploring the path to a pivotal clinical trial

**DOI:** 10.3389/fimmu.2024.1470677

**Published:** 2024-10-01

**Authors:** Cassandra Bazile, Magdy M. Abdel Malik, Courtney Ackeifi, Randy L. Anderson, Roy W. Beck, Marc Y. Donath, Sanjoy Dutta, Joseph A. Hedrick, Stephen R. Karpen, Thomas W. H. Kay, Thomas Marder, Marjana Marinac, Jennifer McVean, Robert Meyer, Jeremy Pettus, Teresa Quattrin, Ruud H. J. Verstegen, Joshua A. Vieth, Esther Latres

**Affiliations:** ^1^ Breakthrough T1D (formerly known as JDRF), New York, NY, United States; ^2^ Quaestio Global Partners, LLC, Chester, NJ, United States; ^3^ Diabetes Clinical Development, Wilmington, NC, United States; ^4^ Jaeb Center for Health Research, Tampa, FL, United States; ^5^ Clinic of Endocrinology, Diabetes and Metabolism, University of Basel, Basel, Switzerland; ^6^ Critical Path Institute, Tucson, AZ, United States; ^7^ St Vincent’s Institute of Medical Research, Fitzroy, VIC, Australia; ^8^ Putnam Associates, New York, NY, United States; ^9^ Medtronic, Northridge, CA, United States; ^10^ Greenleaf Health, Washington, DC, United States; ^11^ Division of Endocrinology and Metabolism, Department of Medicine, University of California San Diego, La Jolla, CA, United States; ^12^ Department of Pediatrics, Jacobs School of Medicine and Biomedical Sciences, University at Buffalo, Buffalo, NY, United States; ^13^ Division of Clinical Pharmacology and Toxicology, Department of Pediatrics, Hospital for Sick Children, Toronto, ON, Canada; ^14^ Division of Rheumatology, Department of Pediatrics, Hospital for Sick Children, Toronto, ON, Canada

**Keywords:** T1D, autoimmunity, disease modifying therapies, TNF-α, clinical trial

## Abstract

Type 1 diabetes (T1D) is an autoimmune disease characterized by the destruction of insulin-producing β-cells in the pancreas. This destruction leads to chronic hyperglycemia, necessitating lifelong insulin therapy to manage blood glucose levels. Typically diagnosed in children and young adults, T1D can, however, occur at any age. Ongoing research aims to uncover the precise mechanisms underlying T1D and to develop potential interventions. These include efforts to modulate the immune system, regenerate β-cells, and create advanced insulin delivery systems. Emerging therapies, such as closed-loop insulin pumps, stem cell-derived β-cell replacement and disease-modifying therapies (DMTs), offer hope for improving the quality of life for individuals with T1D and potentially moving towards a cure. Currently, there are no disease-modifying therapies approved for stage 3 T1D. Preserving β-cell function in stage 3 T1D is associated with better clinical outcomes, including lower HbA1c and decreased risk of hypoglycemia, neuropathy, and retinopathy. Tumor Necrosis Factor alpha (TNF-α) inhibitors have demonstrated efficacy at preserving β-cell function by measurement of C-peptide in two clinical trials in people with stage 3 T1D. However, TNF-α inhibitors have yet to be evaluated in a pivotal trial for T1D. To address the promising clinical findings of TNF-α inhibitors in T1D, Breakthrough T1D convened a panel of key opinion leaders (KOLs) in the field. The workshop aimed to outline an optimal clinical path for moving TNF-α inhibitors to a pivotal clinical trial in T1D. Here, we summarize the evidence for the beneficial use of TNF-α inhibitors in T1D and considerations for strategies collectively identified to advance TNF-α inhibitors beyond phase 2 clinical studies for stage 3 T1D.

## 1 Introduction

Type 1 diabetes (T1D) is an autoimmune disease characterized by an immune-mediated loss of pancreatic β-cells, resulting in insulin deficiency and lifelong insulin dependence. While exogenous insulin therapy is the only live-saving established treatment for T1D, people with T1D require constant proactive management of blood glucose levels in relation to meals and activity despite technological advances in insulin delivery (pumps) and glycemic monitoring (sensors). Moreover, life expectancy is still decreased compared to persons without T1D due to the development of chronic and acute complications, causing considerable morbidity and premature mortality (1, 2). Greater knowledge of immune mechanisms of autoimmune conditions has spurred significant progress in developing and receiving approval for disease-modifying therapies (DMTs) in other autoimmune conditions. However, despite an increase in the prevalence of T1D, the application of DMTs in T1D has lagged compared to other autoimmune diseases. DMTs designed to safeguard functional β-cell mass through controlling/modifying autoimmunity hold promise to slow or halt the course of T1D. Importantly, while autoimmunity and loss of β-cell function, as measured by C-peptide and insulin levels, starts in the pre-symptomatic stages, preservation of remaining β-cell function at the time of clinical diagnosis can provide clinical benefits such as reductions in hypoglycemia, neuropathy and retinopathy (3).

Tumor necrosis factor alpha (TNF-α) inhibitors have demonstrated efficacy in phase 1 and 2 clinical trials for Stage T1D (4, 5). However, they have yet to be tested in a pivotal trial for T1D. In fact, despite promising clinical findings of TNF-α inhibitors and other DMTs in T1D, fewer than 15% of T1D immunotherapies transition from phase 2 to phase 3 clinical trials in stage 3 T1D (4–12). To address the promising clinical findings of TNF-α inhibitors in T1D and the limited transition of DMTs to pivotal clinical trials, Breakthrough T1D convened a panel of key opinion leaders (KOLs) in the field. The workshop goal was to outline an optimal clinical path for moving TNF-α inhibitors to a pivotal clinical trial in T1D. The workshop was held in November 2023, and brought together KOLs representing drug development, autoimmune pathobiology, experience with TNF-α in T1D and other indications, and clinical trial design expertise. The workshop aimed at 1) identifying challenges associated with getting TNF-α inhibitors in the clinical setting for people with T1D, 2) establishing consensus on an optimal path for TNF-α inhibitors from phase 2 studies to phase 3 and FDA approval in people with stage 3 T1D, and 3) identifying commercial opportunities for TNF-α inhibitors. Based on the workshop, this perspective highlights evidence for the beneficial use of TNF-α inhibitors in T1D and outlines considerations and strategies collectively identified to advance TNF-α inhibitors beyond phase 2 clinical studies for stage 3 T1D.

## 2 Current state of clinical trials in stage 3 T1D

Breakthrough T1D’s role in defining distinctive T1D stages has enabled significant advancements in understanding T1D pathogenesis and assessing stage-specific interventions (13–16). Several therapeutic opportunities exist across different stages of disease progression in T1D, spanning from primary prevention upon the detection of autoantibodies (stage 1), to intervention following dysglycemia (stage 2), or therapy post-clinical onset (stage 3) (33). Teplizumab (Tzeild), a humanized anti-CD3 monoclonal antibody, is the first and so far, only DMT approved for adults and children aged 8 and older stage 2 T1D to delay the onset of stage 3 T1D. Currently there are no DMTs approved for stage 3 T1D. Many DMTs that target the adaptive and innate arms of the immune system have been clinically evaluated to treat stage 3 T1D and show promise in preserving β-cell function. While cellular components of the immune system are considered key contributors to β-cell destruction that leads to clinical diagnosis T1D, proinflammatory cytokines play a crucial role in initiating and propagating autoimmunity. TNF-α represents one cytokine that plays a critical role in T1D and can be targeted by established and effective drugs.

## 3 Role of TNF-α driven inflammatory processes in T1D

TNF-α is a pro-inflammatory cytokine implicated in the pathogenesis of several autoimmune conditions. Produced by activated macrophages, dendritic cells (DC), neutrophils, CD4+ lymphocytes, mast cells, eosinophils, neurons, and natural killer cells, TNF-α initiates a cascade of responses including the production of IL-1β and IL-6, enhanced expression of adhesion molecules, and activation of apoptotic and cytotoxic responses (17, 18).

TNF-α has been identified as a critical regulator in the progression of T1D (19–21). Initially, it was observed to enhance antigen presentation, thereby expediting the killing of β-cells by CD8+ T cells in the nonobese diabetic (NOD) mouse model (17). Subsequent investigations using NOD mice revealed TNF-α’s role in promoting the maturation of DCs, particularly within the CD11b+CD11c+ subset, which possessed the ability to activate islet-specific T cells in pancreatic lymph nodes (18). Moreover, direct harm to pancreatic β-cells by TNF-α has been documented, with elevated concentrations of this cytokine implicated in the pathogenesis of T1D in humans (19, 20, 22). Notably, a recent large-scale immune profiling study revealed the enrichment of TNF-α target genes in memory CD4+ T cells in the pancreatic lymph nodes of T1D donors. Interestingly, the same observation was made for non-diabetic antibody-positive donors that have yet progressed to T1D onset, highlighting the potential benefit of using TNF-α inhibitors in earlier stages of T1D. Together these findings suggest in humans that inhibition of TNF-α signaling suppresses memory CD4 T cell activation, which likely results in reduced CD8 T cell-mediated destruction of β cells (23). Given TNF-α’s detrimental role in autoimmune disease, targeting its removal or neutralization is a promising therapeutic strategy.

## 4 The current landscape of TNF-α inhibitors in autoimmune disease

**Figure 1:**
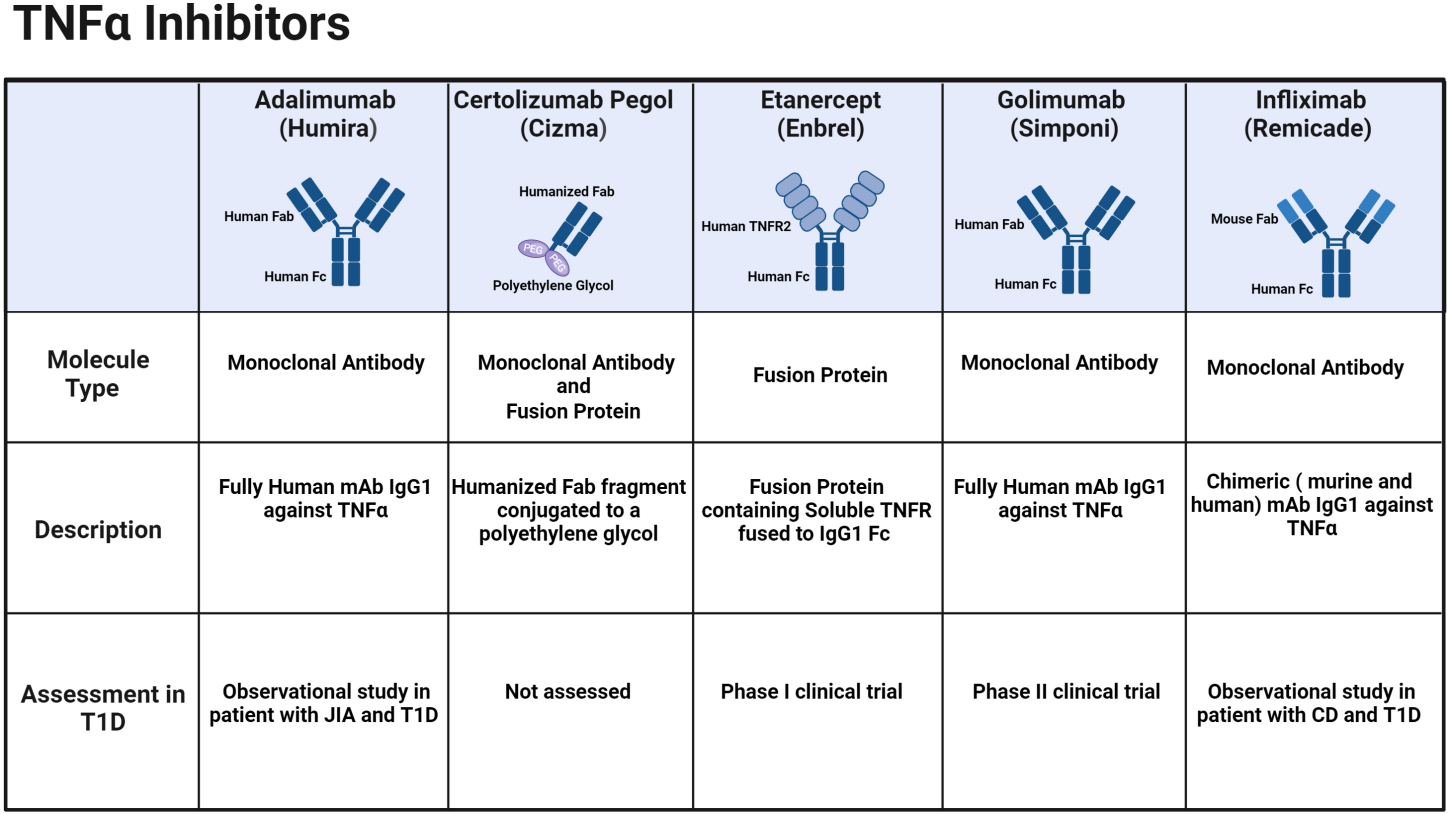
Currently approved TNF-α inhibitors and their molecular type.

TNF-α inhibitors disrupt the binding of TNF-α and its receptors, effectively inhibiting the cytokine-driven inflammatory response. These inhibitors effectively alleviate several autoimmune conditions shown in **Table 1**. The efficacy and safety of both approved TNF-α inhibitors and their corresponding biosimilars have been thoroughly investigated and documented in existing literature including several systematic reviews and meta-analysis of published randomized controlled trials (24–27). There are five branded biologic agents FDA-approved for other autoimmune indications: adalimumab, certolizumab pegol, etanercept, golimumab, and infliximab (**Figure 1**; **Table 1**). Additionally, sixteen biosimilar TNF-α inhibitors have been granted FDA approval, broadening the array of available treatment options. Although these inhibitors share common indications, each agent also has unique properties and applications that vary based on the specific indication, for example the route of administration—subcutaneous or intravenous. —The FDA-approved indications for each of the TNF-α inhibitors brands and their biosimilars are summarized below (**Table 1**).

**Table 1:** Approved Biologic and Biosimilar TNF-α Inhibitors and their therapeutic indications: Rheumatoid Arthritis (RA), Juvenile Idiopathic Arthritis (JIA), Psoriatic Arthritis (PsA), Ankylosing Spondylitis (AS), Adult Crohn’s Disease (ACD), Pediatric Crohn’s Disease (PCD), Ulcerative Colitis (UC), Pediatric Ulcerative Colitis (PUC), Plaque Psoriasis (Ps), Hidradenitis Suppurativa (HS) and Uveitis (UV).

TNF-α Inhibitors/Brands	Biosimilars	Marketers	Approval Date	Approved Indications										
				**RA**	**JIA**	**PsA**	**AS**	**ACD**	**PCD**	**UC**	**PUC**	**Ps**	**HS**	**UV**
**Adalimumab/Humira**		AbbVie Inc.	2002	X	X	X	X	X	X	X		X	X	X
	Abrilada	Pfizer Inc.	2019	X	X	X	X	X		X		X		
	Amjevita	Amgen Inc.	2016	X	X	X	X	X		X		X		
	Cyltezo	Boehringer Ingelheim GmbH	2021	X	X	X	X	X		X		X		
	Hadlima	Merck & Co	2019	X	X	X	X	X		X		X		
	Hulio	Mylan (Viatris)	2020	X	X	X	X	X		X		X		
	Hyrimoz	Sandoz AG/Novartis AG	2018	X	X	X	X	X		X		X		
	Yusimry	Coherus BioSciences	2021	X	X	X	X	X	X	X		X		
	Idacio	Fresenius Kabi	2022	X	X	X	X	X	X	X		X		
	Simlandi	Teva Pharmaceuticals	2024	X	X	X	X	X		X		X	X	X
	Yuflyma	Celltrion	2023	X	X	X	X	X	X	X		X	X	
**Certolizumab pegol/Cimzia**		UCB Inc.	2008	X		X	X	X						
**Etanercept/Enbrel**		Immunex/Pfizer & Amgen	1998	X	X	X	X					X		
	Erelzi	Sandoz Inc/Novartis Inc.	2016	X	X	X	X					X		
	Eticovo	Samsung Bioepis Co	2019	X	X	X	X					X		
**Golimumab/Simponi**		Janssen Biotech, Inc.	2009	X		X	X							
**Golimumab/Simponi Aria**		Janssen Biotech, Inc.	2009	X	X	X	X							
**Infliximab/Remicade**		Janssen Biotech, Inc.	1998	X		X	X	X	X	X	X	X		
	Avsola	Amgen Inc	2019	X		X	X	X	X	X	X	X		
	Inflectra	Hospira/Pfizer Inc.	2016	X		X	X	X	X	X	X	X		
	Ixifi	Pfizer Inc.	2017	X		X	X	X	X	X		X		
	Renflexis	Samsung Bioepis/Merck & Co	2017	X		X	X	X	X	X		X		

"X" indicates FDA- approved indications for each TNF inhibitor brand or bio-similar.

## 5 Clinical trials and case studies using TNF-α inhibitors in T1D

Observational studies on IBD treatment in T1D patients have demonstrated the benefits of TNF-α inhibitors in improving hypoglycemia and insulin resistance in persons with established (stage 4) T1D. A 29-year-old man with T1D saw improvements in glycemic control, reduced hypoglycemic episodes, and significant changes in insulin secretion and resistance upon starting infliximab treatment (28). However, a case report highlighted erratic blood sugar levels and severe hypoglycemia in a patient with well-controlled T1D within 12 hours of adalimumab treatment (29). Several other case reports noted improved glycemic control with adalimumab, though occasionally accompanied by hypoglycemic episodes (30, 31).

Importantly, two clinical trials have shown the benefit of using TNF-α inhibitors to preserve β-cell function in children with stage 3 T1D. In a small, 24-week double-masked, randomized, placebo-controlled phase 1 clinical trial, etanercept was assessed in 18 new-onset pediatric patients ages 3-18. At 24 weeks, HbA1C was reduced, C-peptide was increased, and insulin dose was lower in the etanercept-treated group compared to the placebo (4). Following this study, a phase 2 placebo-controlled, double-blind study assessed the use of golimumab in 84 children ages 6-21 in Stage 3 T1D. Golimumab, administered bi-weekly for 52 weeks, resulted in better maintenance of endogenous insulin production and less exogenous insulin use than placebo (5). Furthermore, 12 months after stopping therapy the golimumab group showed persistently higher C-peptide compared to the placebo group (32).

## 6 Discussion: strategies for the advancement of TNF-α inhibitors for T1D

Studies across 11 autoimmune indications have largely informed our understanding of TNF-α inhibitors as therapeutic agents. Given there are 5 FDA-approved branded TNF-α inhibitors, our workshop focused mainly on approved indications and clinical studies in pediatric populations with safety data (**Table 1**). During the discussion, workshop participants identified regulatory and commercial considerations and potential strategies for advancing TNF-α inhibitors to registrational trials and regulatory approval.

Regulatory Considerations: Despite phase 2 trials demonstrating the efficacy of certain immune therapies for T1D, these treatments have not yet received approval for use in stage 3 T1D. The focus on glycemic control to evaluate the success of DMTs in stage 3 T1D poses challenges for sponsors, as changes in clinical parameters and long-term complications develop gradually, necessitating lengthy trials to measure directly. Additionally, concurrent insulin replacement therapy makes assessing the effects on HbA1c without extensive trials challenging. DMTs aim to preserve beta cell function, with current T1D trials relying on markers such as C-peptide to gauge their impact on beta cells. However, regulators have indicated that C-peptide alone is insufficient to demonstrate clinical benefits and cannot serve as the sole primary endpoint for traditional or full product approval. C-peptide levels decline rapidly after diagnosis, allowing for assessing a treatment’s effects within trials of more manageable size and duration. Thus, recognizing the clinical significance of C-peptide offers the potential to expedite the development and approval of DMTs for stage 3 T1D. The TOMI-T1D (Trial Outcome Markers Initiative-T1D) meta-analysis of 21 previous trials of DMTs in stage 3 T1D demonstrated that DMTs that effectively preserve β cell function, as measured by C-peptide, were also associated with consistent reductions in HbA1c over the course of the clinical trials (15). Furthermore, in a recent publication, a Breakthrough T1D-led perspective outlined a significant body of evidence from 3 datasets demonstrating that even small amounts of preserved endogenous insulin production, assessed as C-peptide in people with T1D are linked to better clinical outcomes, including lower HbA1c, decreased risk of hypoglycemia, neuropathy, and retinopathy (3). C-peptide is not currently accepted by regulators when used alone as the basis of approval, and if it were accepted as a validated endpoint it could lead to accelerated clinical trials in people with stage 3 T1D.

Moving forward, validation of biomarkers for immunotherapy efficacy in T1D should provide insight into glycemic control, β-cell health, and immune regulation. Recent focus on “β-cell death” assays and proinsulin levels alongside C-peptide and insulin measurements represent a promising approach (34–36). Additionally, being able to identify non-responders would be transformative, aiding in tailored interventions and a personalized medicine approach. A recent study analyzing samples from a phase 2 clinical trial assessing Golimumab in stage 3 T1D showed that baseline metabolites and miRNAs best predicted β cell function for both placebo and treatment arms (37). Studies to determine predictive biomarkers of response in patients with inflammatory bowel disease (IBD) and rheumatoid arthritis (RA) treated with TNF-α inhibitors are still developing; however, recent work has revealed potential biomarkers of response such as oncostatin M and increased switched memory B cells, respectively (38–40). Intriguingly, a novel computational approach termed disruption networks revealed in a recent study that the RAC1-PAK1 signaling cascade is associated with TNF-α inhibitor response in RA and IBD patients. A similar approach may warrant use in T1D, considering that biomarkers analyzed at baseline and post-treatment could aid in stratifying subjects likely to respond to treatment and aid in the design of adaptive trials where non-responders could receive alternative interventions (13, 37). Regulatory agency consultation and biomarker utilization from T1D clinical trials and other autoimmune diseases are crucial for informed trial designs, ensuring safety and efficacy.

Commercial Considerations: No further investigations have been reported by the owners of the etanercept and golimumab assets since the early publications (4, 5, 32). This posture stems partly from the fact that T1D is infrequent compared to other chronic disorders thus limiting the return on investment coupled with of the complex pathogenesis of T1D and regulatory considerations.

Moreover, there is a widespread perception and acceptance of insulin as a satisfactory treatment. T1D is a complex metabolic disorder necessitating a comprehensive treatment regimen. Current T1D management is almost entirely insulin-centric. While insulin therapy remains fundamental, it often fails to achieve optimal glycemic and metabolic control. T1D poses numerous challenges beyond insulin deficiency, including under-appreciated pathophysiologies, such as severe dysregulation of glucagon action and the absence of the metabolic hormone amylin. Many affected people exhibit metabolic imbalances such as hyperketonemia, insulin resistance, and obesity (41, 42). Finally, depression and other mental health challenges are common in people with T1D; for example, in the T1D Exchange clinic registry, it was found that up to 10 percent of adults had probable major depression, which correlated with worsened diabetes outcomes such as elevated HbA1c and diabetic ketoacidosis (DKA) events (43).

Furthermore, limited interest in expanding T1D as an indication is partially due to its association with a broad spectrum of autoimmune diseases, some of which have larger markets and primarily affect adults. Consequently, drug development is often prioritized in these diseases when advancing new drugs, in the autoimmune spectrum of disease. By the time T1D becomes a focus, the drug may be approaching the end of its patent life, prompting companies to shift their focus to developing new drugs rather than exploring additional indications for existing ones. Moreover, while T1D is a priority for large pharmaceutical companies there is emphasis on the larger market represented by type 2 diabetes.

Etanercept biosimilars (erelzi and eticovo) received FDA approval in 2016 and 2019, respectively, for the same indications as etanercept, as outlined in **Table 1**. It is worth mentioning, that neither biosimilar of the etanercept/enbrel has commercially launched in the US due to patent challenges, and they are not expected to until 2029. Although not yet studied in clinical trials for T1D, these biosimilars are likely to yield similar outcomes to etanercept. Currently, there are no FDA-approved biosimilars for golimumab. Generally, biosimilars are approved by the FDA for the same indications as the original biologics. However, the manufacturers of these biosimilars can potentially explore new indications such as T1D.

Opportunities for advancement: Many opportunities exist for the advancement of TNF-α inhibitors for treating T1D. TNF-α inhibitors are a well-established and safe treatment for pediatric populations, with adalimumab, infliximab, golimumab and etanercept having FDA-approved indications for children (**Table 1**). TNF-α inhibitors with known safety profiles in pediatric populations offer potential use in stage 3 T1D, given that roughly 50% of new-onset T1D cases occur in children and adolescents (44). In a phase 3 clinical trial, teplizumab was assessed in 318 newly diagnosed children 8-17 (45). This trial provides a framework that could be applied to determining TNF-α inhibitors in a pivotal clinical trial. Currently, most trials for T1D DMTs are fixed designs, but there is a shift toward using adaptive designs for more significant time and cost efficiency. Adaptive clinical trials allow *a priori* defined design changes based on accumulating data. Commercial interest in adaptive designs has risen since the FDA’s 2019 guidance for adaptive trials. For example, a two-stage group-sequential design with interim analysis after the first stage can provide information to support interim business decisions while maintaining the trials scientific integrity for the second stage efficacy analysis.

Anti-cytokine therapies such as TNF-α inhibitors are well-tolerated treatments for autoimmunity, making them attractive choices for synergistic dual therapy with other immune or β-cell regeneration therapies. Combining TNF-α inhibitors in a staggered approach with a therapy that targets or depletes adaptive immune cells may lead to positive clinically relevant measures such as lessened daily insulin requirements and β cell preservation as measured by C-peptide. Evaluation of TNF-α inhibitors in stage 2 T1D also represents a potential path for moving TNF-α inhibitors to the clinical setting and may even support testing TNF-α inhibitors in combination with other approved DMTs. Furthermore, insights garnered from the development of TNF-α-blocking biologic therapies for autoimmune indications such as RA could be mirrored. While this may not perfectly translate to T1D, there should be an emphasis on insights derived from humans and mouse models. This involves utilizing *in vitro* assays, isogenic cellular systems, NOD mice, humanized mouse models, and cadaveric samples to validate proof of concept and explore underlying mechanisms (37).

In summary, there is an unmet medical need for treatments that address the root cause of diseases rather than replacing the loss of βcell function with an exogenous replacement therapy that requires constant monitoring of glucose levels and a dosing regimen of insulin, particularly in the case of T1D. Despite advancements in diabetes management, such as improved insulin delivery systems, many individuals fail to achieve optimal glycemic control, causing complications and a significant burden. Preserving endogenous ß-cell function is crucial not only for insulin independence but also for preventing long-term complications.

TNF-α inhibitors have demonstrated maintenance of C-peptide and reduced insulin use at the time of clinical diagnosis, representing a pivotal autoimmune intervention currently used in numerous conditions that shows broad and lasting treatment success and has FDA approval for long-term use in pediatric populations. While many DMTs have shown efficacy in phase 2 clinical trials in stage 3 T1D, we selected TNF-α inhibitors as an asset to forge a path for progressing DMTs to a phase 3 clinical trial. TNF-α inhibitors have demonstrated a strong safety and efficacy profile in 11 autoimmune diseases, with approvals for pediatrics and adult indications. Furthermore, five distinct biologics and 16 biosimilars are commercially available. Given the promising clinical trial data in two stage 3 T1D clinical trials, assessing TNF-α inhibitors in a pivotal trial is a feasible and substantiated initiative. Despite existing challenges, registrational clinical trials using adaptive designs or combination approaches that couple TNF-α inhibitors with an agent with a different mechanism of action represent an opportunity to advance TNF-α inhibitors for treating people with stage 3 T1D. Breakthrough T1D remains optimistic about the potential benefits of TNF-α inhibitors in significantly impacting individuals affected by T1D. We invite commercial developers and the research community to aid in the design of strategies to advance TNF-α inhibitors to a pivotal clinical trial for stage 3 T1D.

## Data Availability

The original contributions presented in the study are included in the article/supplementary material. Further inquiries can be directed to the corresponding author.
